# Multiplex genetic manipulations in *Clostridium butyricum* and *Clostridium sporogenes* to secrete recombinant antigen proteins for oral-spore vaccination

**DOI:** 10.1186/s12934-024-02389-y

**Published:** 2024-04-24

**Authors:** Yanchao Zhang, Tom S. Bailey, Philip Hittmeyer, Ludwig J. Dubois, Jan Theys, Philippe Lambin

**Affiliations:** 1 The M-Lab, Department of Precision Medicine, GROW - Research Institute for Oncology and Reproduction, Maastricht University, Maastricht, 6229 ER the Netherlands; 2 Department of Cell Biology–Inspired Tissue Engineering, MERLN Institute for Technology-Inspired Regenerative Medicine, Maastricht University, Maastricht, 6229 ER the Netherlands; 3 LivingMed Biotech BV, Clos Chanmurly 13, Liège, 4000 Belgium

**Keywords:** Clostridia, Genetic manipulation, Protein secretion, AUU start codon, NY-ESO-1/CTAG1, Spike_S1

## Abstract

**Background:**

*Clostridium* spp. has demonstrated therapeutic potential in cancer treatment through intravenous or intratumoral administration. This approach has expanded to include non-pathogenic clostridia for the treatment of various diseases, underscoring the innovative concept of oral-spore vaccination using clostridia. Recent advancements in the field of synthetic biology have significantly enhanced the development of *Clostridium*-based bio-therapeutics. These advancements are particularly notable in the areas of efficient protein overexpression and secretion, which are crucial for the feasibility of oral vaccination strategies. Here, we present two examples of genetically engineered *Clostridium* candidates: one as an oral cancer vaccine and the other as an antiviral oral vaccine against SARS-CoV-2.

**Results:**

Using five validated promoters and a signal peptide derived from *Clostridium sporogenes*, a series of full-length NY-ESO-1/CTAG1, a promising cancer vaccine candidate, expression vectors were constructed and transformed into *C. sporogenes* and *Clostridium butyricum*. Western blotting analysis confirmed efficient expression and secretion of NY-ESO-1 in clostridia, with specific promoters leading to enhanced detection signals. Additionally, the fusion of a reported bacterial adjuvant to NY-ESO-1 for improved immune recognition led to the cloning difficulties in *E. coli*. The use of an AUU start codon successfully mitigated potential toxicity issues in *E. coli*, enabling the secretion of recombinant proteins in *C. sporogenes* and *C. butyricum*. We further demonstrate the successful replacement of PyrE loci with high-expression cassettes carrying NY-ESO-1 and adjuvant-fused NY-ESO-1, achieving plasmid-free clostridia capable of secreting the antigens. Lastly, the study successfully extends its multiplex genetic manipulations to engineer clostridia for the secretion of SARS-CoV-2-related Spike_S1 antigens.

**Conclusions:**

This study successfully demonstrated that *C. butyricum* and *C. sporogenes* can produce the two recombinant antigen proteins (NY-ESO-1 and SARS-CoV-2-related Spike_S1 antigens) through genetic manipulations, utilizing the AUU start codon. This approach overcomes challenges in cloning difficult proteins in *E. coli*. These findings underscore the feasibility of harnessing commensal clostridia for antigen protein secretion, emphasizing the applicability of non-canonical translation initiation across diverse species with broad implications for medical or industrial biotechnology.

**Supplementary Information:**

The online version contains supplementary material available at 10.1186/s12934-024-02389-y.

## Background

The *Clostridium* genus, characterized by its obligate anaerobic nature, endospore formation, and Gram-positive cell wall-secreting extracellular proteins [1], gained early attention in cancer therapy in 1813 with the observation of tumour regression induced by *Clostridium perfringens* [2]. Subsequently, the specific colonization of *Clostridium*, particularly *Clostridium novyi*, in tumour necrotic regions has offered a unique avenue in cancer therapy, a phenomenon validated through recent pre-clinical and clinical trials [3–6]. Clostridia-directed enzyme prodrug therapy (CDEPT) has emerged as a promising strategy, demonstrating significant enhancement in tumour regression through the administration of a nontoxic prodrug in vivo [7–10]. Concurrently, the innovative approach of genetically engineering clostridia for the production of cytokines or antibodies, termed *Clostridium*-mediated cancer immunotherapy (CMIT), has further expanded the therapeutic potential of clostridia in cancer treatment [11, 12].

Beyond oncology, research interests in commensal *Clostridium* spp. have broadened to encompass the exploration of microbe-host interactions and biomedical applications [13]. *Clostridium* oral-spore vaccination (COSV) stands out as a notable concept, capitalizing on the highly resistant endospores and anaerobic germination in the small intestine [14]. This interest in engineering non-pathogenic clostridia will extend to various disease treatments.

Advancements in *Clostridium* synthetic biology tools have played a pivotal role in the development of *Clostridium*-based bio-therapeutics (CBT) [15, 16]. Genetic manipulation of oncological *Clostridium sporogenes* or probiotic *Clostridium butyricum* has resulted in the production of bioactive interleukin-2 (IL-2) [17, 18], granulocyte macrophage-colony stimulating factor (GM-CSF) [19], porcine epidermal growth factor (pEGF) [20], and glucagon-like peptide-1 (GLP-1) [21, 22]. Despite these achievements, challenges persist in the expression of heterologous proteins within clostridia due to issues such as protein toxicity and cloning difficulty observed in *E. coli* cloning strains, a necessary intermediate host for molecular biology cloning. Consequently, the implementation of well-regulated expression systems becomes imperative. Existing inducible expression systems, including lactose [23] or tetracycline-inducible [24] and bacteriophage T7 systems [25], have exhibited promise across *E. coli* and clostridia species. However, their application may lead to undesired expression levels for CBT [26]. Thus, continued exploration of orthogonal control mechanisms for regulating protein overexpression across different species remains essential to propelling the development of CBT.

For the concept of COSV, the New York esophageal squamous cell carcinoma 1 (NY-ESO-1, also known as CTAG1), a well-established cancer-testis antigen, is a potentially promising target for oral cancer vaccines due to its re-expression in various cancer types [27]. The spike proteins of SARS-CoV-2 are the main targets of COVID-19 vaccines [28] and consequently also serve as potential targets for antiviral oral vaccines against SARS-CoV-2. In addition, a bacterial adjuvant of chimeric designer peptide (CDP) has been demonstrated to elicit superior immune responses against SARS-CoV-2 [29]. In this study, we hypothesized that multiplex genetic manipulations could enable efficient overexpression and secretion of antigen proteins in clostridia. Our objective was to demonstrate the feasibility of this hypothesis by engineering two gut commensal *Clostridium* spp., *C. butyricum* and *C. sporogenes*, to secrete the NY-ESO-1 cancer neo-antigen and the recombinant SARS-CoV-2-related Spike_S1 antigens, conjugated with the reported adjuvant of the CDP. Our results underscore the ability of clostridia to utilize the AUU start codon for initiating translation and secreting proteins.

## Methods

### Bacterial strains and cultivation conditions

Details of the bacterial strains employed in this study are summarized in Table 1. For vector cloning and bacterial conjugation, two distinct *Escherichia coli* strains, namely 10-β and S17-1, were utilized. All *E. coli* strains were cultivated aerobically in lysogeny broth (LB) at 37 °C. When applicable, the growth medium was supplemented with chloramphenicol (Cm) at concentrations ranging from 12.5 to 25 µg/mL.

**Table 1:** Strains and plasmids used in this work

	Description	Sources
**Strains**		
*E. coli* 10-β	High efficiency strain ideal for cloning	C3019, NEB
*E. coli* S17-1	Conjugative donor strain	ATCC 47,055
*C. sporogenes* WT	Wild type strain, *Clostridium sporogenes* NCIMB 10,696	NCIMB culture collection (UK)
C. *butyricum* WT	Wild type strain, *Clostridium butyricum* DSM10702	DSMZ-German Collection
*C. butyricum*Δ*pyrE*	*C. butyricum* with deletion of the PyrE loci	[28]
*C. butyricum*IN*pyrE*::ESO	*C. butyricum* with integration of the miniP*c.b*_fU-*NY-ESO-1* into the PyrE loci	This work
*C. butyricum*IN*pyrE*::CDP-ESO	*C. butyricum* with integration of the miniP*c.b*_fU-CDP-*NY-ESO-1* into the PyrE loci	This work
*C. sporogenes*-NT	Non-toxic *C. sporogenes* NCIMB 10,696, via deletion of the putative Streptolysin S operon	[18]
*C. sporogenes*-NTΔ*pyrE*	*C. sporogenes*-NT with deletion of the PyrE loci	This work
*C. sporogenes*-NTIN*pyrE*::ESO	*C. sporogenes*-NT with integration of the miniP*c.sp*_fU-*NY-ESO-1* into the PyrE loci	This work
*C. sporogenes*-NTIN*pyrE*::CDP-ESO	*C. sporogenes*-NT with integration of the miniP*c.sp*_fU-CDP-*NY-ESO-1* into the PyrE loci	This work
*C. sporogenes*-NTIN*pyrE*::*S1*	*C. sporogenes*-NT with integration of the miniP*c.b*_fU-*S1* into the PyrE loci	This work
*C. sporogenes*-NTIN*pyrE*::CDP-*S1*	*C. sporogenes*-NT with integration of the miniP*c.b*_fU-CDP-*S1* into the PyrE loci	This work
**Plasmids**		
pGG2121	Golden Gate assembling and *E. coli* - *Clostridium* shuttle vector, Cm^r^	[27]
pP*romoters*-Sp*nprM3*-*NY-ESO-1*	pGG2121 ligated with promoters, the *nprM3* signal peptide, and the codon-optimized *NY-ESO-1* gene	This work
pP*romoters*-Sp*nprM3*-CDP-*NY-ESO-1*	pGG2121 ligated with promoters, the *nprM3* signal peptide, and the codon-optimized CDP sequence and *NY-ESO-1* gene	This work
pP*romoters*-Sp*nprM3*-*S1*	pGG2121 ligated with promoters, the *nprM3* signal peptide, and the codon-optimized *Spike_S1* sequence	This work
pP*romoters*-Sp*nprM3*-CDP-*S1*	pGG2121 ligated with promoters, the *nprM3* signal peptide, and the codon-optimized CDP and *Spike_S1* gene sequence	This work
pP*IPL12*-FnCas12a	Entry vector with tetracycline-Inducible expression of FnCas12a in *E. coli* and clostridia, Erm^r^	[28]
pP*fet*Fn-Target_v2	Entry vector with gRNA expression for FnCas12a in *E. coli* and clostridia, Cm^r^	[28]
pP*fet*Fn-IncbPyrE::ESO	pP*fet*Fn-Target_v2 ligated with the donor DNA template and target for integrating the miniP*c.b*_fU-*NY-ESO-1* expression cassette into *C. butyricum* PyrE loci	This work
pP*fet*Fn-IncbPyrE::CDP-ESO	pP*fet*Fn-Target_v2 ligated with the donor DNA template and target for integrating the miniP*c.b*_fU-CDP-*NY-ESO-1* expression cassette into *C. butyricum* PyrE loci	This work
pP*fet*Fn-DspPyrE	pP*fet*Fn-Target_v2 ligated with the donor DNA template and target for deleting *pyrE* gene in *C. sporogenes*-NT	This work
pP*fet*Fn-InspPyrE::ESO	pP*fet*Fn-Target_v2 ligated with the donor DNA template and target for integrating the miniP*c.sp*_fU-*NY-ESO-1* expression cassette into *C. sporogenes*-NT PyrE loci	This work
pP*fet*Fn-InspPyrE::CDP-ESO	pP*fet*Fn-Target_v2 ligated with the donor DNA template and target for integrating the miniP*c.sp*_fU-CDP-*NY-ESO-1* expression cassette into *C. sporogenes*-NT PyrE loci	This work
pP*fet*Fn-InspPyrE::S1	pP*fet*Fn-Target_v2 ligated with the donor DNA template and target for integrating the miniP*c.b*_fU-*S1* expression cassette into *C. sporogenes*-NT PyrE loci	This work
pP*fet*Fn-InspPyrE::CDP-S1	pP*fet*Fn-Target_v2 ligaed with the donor DNA template and target for integrating the miniP*c.b*_fU-CDP-*S1* expression cassette into *C. sporogenes*-NT PyrE loci	This work

*Clostridium* strains (*C. sporogenes* NCIMB 10,696 and *C. butyricum* DSM10702) were cultured under strict anaerobic conditions at 37 °C (MG1000 Mark II, 27 Don Whitley, UK; 80% N2, 10% CO2, and 10% H2) in peptone yeast thioglycolate growth medium supplemented with 10 g/L D-glucose (PYTG). Additionally, the medium was supplemented with thiamphenicol (Tm) at concentrations of 15 to 20 µg/mL, erythromycin (Erm) at 15 µg/mL, or D-cycloserine (Cs) at 250 µg/mL, as needed.

### Plasmid construction

Detailed information about the plasmids utilized in this study is presented in Table 1. The *E. coli*-*Clostridium* shuttle vector, pGG2121, was designed for Golden Gate assembly [30]. The colony PCR employed the primer pair M13-F/R for all derivative vectors of pGG2121 (DreamTaq DNA Polymerase, Thermo Scientific). High-fidelity PCR amplifications were carried out using Phusion Plus DNA Polymerase (Thermo Scientific). The primers (Table S1) and gBlock fragments (SP-NY-ESO-1, SP-CDP, and SP-Spike_S1, Table S2) were synthesized by IDT (Integrated DNA Technologies). Plasmid verification was conducted through Sanger sequencing by GENEWIZ.

To determine the most suitable expression cassette, five promoters (P*ptb*, P*thl*_fU, P*fdx*_fU, miniP*c.b*_fU, and miniP*c.sp*_fU) with different strengths were chosen from our previous promoter library and contained BsaI restriction sites [30]. For the construction of NY-ESO-1 expression plasmids, the pGG2121 vector, promoters, and SP-NY-ESO-1 fragment were ligated using Golden Gate assembly (BsaI-HFv2, NEB), resulting in the vector pP*romoters*-Sp*nprM3*-*NY-ESO-1*.

In constructing CDP-NY-ESO-1 expression plasmids, the NY-ESO-1 fragment for CDP fusion (CDP_ESO) was amplified using the primer pair CDP_ESO-F-BsaI/ESO-R-BsaI, and the ATT_SP-CDP fragment with the AUU start codon was amplified using ATT_SP-F-BsaI/CDP-R-BsaI. Subsequently, promoters and CDP_ESO were ligated with SP-CDP or ATT_SP-CDP fragments into pGG2121 using Golden Gate assembly (BsaI-HFv2, NEB), resulting in the vector pP*romoters*-Sp*nprM3*-CDP-*NY-ESO-1*. Analogously, the S1 and CDP-S1 expression vectors (pP*romoters*-Sp*nprM3*-*S1* and pPr*omoters*-Sp*nprM3*-CDP-*S1*) were generated as described above, utilizing the primers in Table S1.

For the construction of genome integration plasmids targeting PyrE loci in clostridia, target fragments were amplified and ligated into the pP*fet*Fn-Target_v2 entry vector of the CRISPR-FnCas12a system [31] using Golden Gate assembly (BsmBI-v2, NEB). After sequencing confirmation, the ligated vectors were digested by AatII and SalI enzymes (NEB), resulting in a 6.5-kb backbone. Subsequently, gene expression cassette fragments were amplified from expression vectors, and upstream and downstream fragments of *C. butyricum* and *C. sporogenes* PyrE loci were amplified using the primers in Table S1. These fragments were then fused using the Golden Gate Assembly (BsaI-HFv2, NEB). The resultant donor DNA template fragments were digested with AatII and SalI enzymes, and the donor DNA templates were ligated to the backbone fragments using T4 DNA ligase (NEB), yielding a series of genome integration plasmids for the CRISPR-FnCas12a system (pP*fet*Fn-IncbPyrE::ESO, pP*fet*Fn-IncbPyrE::CDP-ESO, pP*fet*Fn-DspPyrE, pP*fet*Fn-InspPyrE::ESO, pP*fet*Fn-InspPyrE::CDP-ESO, pP*fet*Fn-InspPyrE::S1, and pP*fet*Fn-InspPyrE::CDP-S1).

All plasmids were introduced into *E. coli* through heat shock, followed by conjugation (*E. coli* S17-1) into clostridia as previously described [31].

### Genome engineering

CRISPR-FnCas12a-mediated genome engineering in clostridia was performed as we previously described [31]. Briefly, the entry vector pP*IPL12*-FnCas12a was conjugated into clostridia, followed by the conjugation of the corresponding genome integration plasmids into these clostridia. Subsequently, trans-conjugants were re-streaked under the induction of 96 ng/mL anhydrotetracycline. Mutations were confirmed through colony PCR, and plasmid-curing with the addition of 100 g/L sucrose was verified by growth under the corresponding antibiotics. All desired mutations were validated by Sanger sequencing.

### Protein sample preparation

For the Western blotting analysis, proteins from *Clostridium* cells and culture supernatants were prepared as follows [32]: *Clostridium* strains were sub-cultured from overnight cultures into fresh media at a 1:50 dilution, with or without the appropriate antibiotics. When the OD_600_ of the cultures reached about 1.0, 1.6 mL of the culture was centrifuged at 12,000 g for 10 min at 4 ºC. The supernatant was transferred to a new tube, and the pellet was frozen at -20 ºC. The supernatant was then treated with 0.03% sodium deoxycholate detergent (Thermo Fisher) and incubated for 10 min at room temperature (RT). Subsequently, the supernatant was incubated with 180 µL of 100% trichloroacetic acid solution (Sigma-Aldrich) for 30 min on ice, and the resultant supernatant precipitation was frozen at -20 ºC. The next day, the pellet was gently incubated with 250 µL of the diluted BugBuster Master Mix/protease solution (1:25) [30] at RT for 30 min. After centrifugation at 12,000 g for 20 min at 4 ºC, the supernatant was extracted into a new tube, serving as the protein sample in the *Clostridium* cell lysate. On the other hand, the supernatant precipitation at -20 ºC was defrosted and centrifuged at 12,000 g for 30 min at 4 ºC. The precipitation was washed three times with 200 µL of 70% ethanol, acetone, and distilled water, respectively (each time centrifuged at 12,000 g for 5 min at 4 ºC). Finally, all water was discarded, and the protein precipitate from the *Clostridium* culture supernatant was ready.

### Western blotting analysis

Before loading onto the Bis-Tris protein gel, the protein samples were incubated in the reduced loading dye [50 mM Tris(2-carboxyethyl)phosphine hydrochloride (TCEP, Thermo Fisher) in 1×LDS sample loading buffer (Thermo Fisher)] for 10 min at RT. For plasmid-based samples, 14 µL of the reduced sample was loaded into each well of NuPAGE 4 to 12% Bis-Tris Mini Protein Gel (NP0323BOX, Thermo Fisher), while for genome-integrated samples, 28 µL was loaded into each well of the NP0327BOX gel (Thermo Fisher). Additionally, 5 µL of PageRuler Prestained Protein Ladder (26,616, Thermo Fisher) served as size standards, and for the Spike_S1 analysis, 50 ng of the recombinant SARS-CoV-2 S1 protein produced in HEK293 cells (HY-P7436, MedChemExpress) was included as the positive control.

The gel was blotted onto a 0.2 μm PVDF membrane using the Trans-Blot Turbo transfer system (1,704,156, Bio-Rad). Subsequently, the membrane was blocked for 1.5 h in a PBS solution with 0.1% Tween 20 and 5% non-fat dry milk (sc-2324, Santa Cruz Biotechnology) at RT, and then was washed three times in the PBS solution with 0.1% Tween 20 for 5 min at RT. Following this, the membrane was incubated overnight at 4 °C with the corresponding antibody diluted at 1:1000 (for NY-ESO-1 related samples, NY-ESO-1 Rabbit mAb, 45,437, Cell Signaling Technology; for Spike_S1 related samples, unless otherwise specified, SARS-CoV-2 S Protein RBD Mouse mAb, 944,804, Biolegend). The membrane was then washed three times in the PBS solution with 0.1% Tween 20 for 15 min at RT, followed by a one-hour incubation step at RT with the corresponding secondary antibody diluted at 1:2000 [Horseradish peroxidase (HRP)-conjugated anti-rabbit IgG antibody, W4011, Promega; HRP-conjugated anti-mouse IgG antibody, 7076, Cell Signaling Technology]. Finally, the membrane was washed three times in the PBS solution with 0.1% Tween 20 for 20 min at RT, and chemiluminescence was detected by incubation with the West Pico PLUS Chemiluminescent Substrate (34,580, Thermo Fisher) and subsequent analysis with the Azure 600 Imaging System (Azure Biosystems).

## Results

### *C. sporogenes* and *C. butyricum* secrete recombinant full-length NY-ESO-1 antigen

In this study, five validated promoters (P*ptb*, P*thl*_fU, P*fdx*_fU, miniP*c.b*_fU, and miniP*c.sp*_fU) and the signal peptide Sp*nprM3* (SP) derived from *C. sporogenes* were fused with the codon-optimized *NY-ESO-1* gene in the *E. coli*-*Clostridium* shuttle vector pGG2121. This resulted in a series of NY-ESO-1 expression vectors named pP*romoters*-Sp*nprM3*-*NY-ESO-1*.

**Fig. 1:**
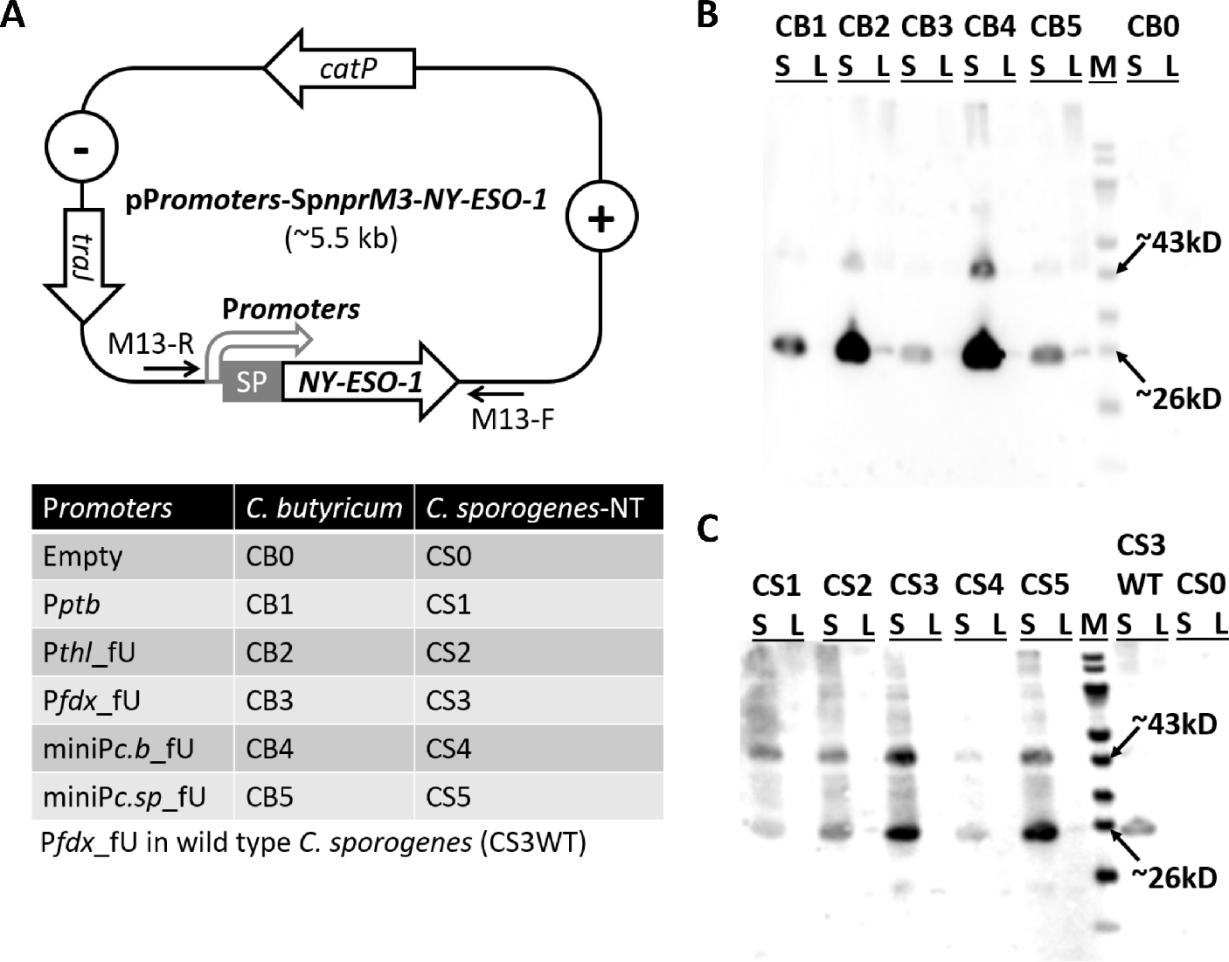
*C. sporogenes* and *C. butyricum* secrete recombinant full-length NY-ESO-1 antigen. (**A**) The schema of the NY-ESO-1 expression plasmid, pP*romoters*-Sp*nprM3*-*NY-ESO-1*, and the details of the used NY-ESO-1 expression clostridia. (−), Gram-negative replicons; (+), Gram-positive replicon; *catP*, thiamphenicol or chloramphenicol resistance gene; *traJ*, conjugal transfer gene; SP, signal peptide Sp*nprM3*; M13-R/F, universal primer pair. Western blotting analysis of NY-ESO-1 expression in *C. butyricum* (**B**) and *C. sporogenes* (**C**). S, culture supernatant; L, pellet lysate; M, PageRuler Prestained Protein Ladder

The constructed vectors, along with the empty vector pGG2121, were subsequently transformed into *C. butyricum* and *C. sporogenes* via conjugation with *E. coli*. *C. butyricum* and non-toxic *C. sporogenes* (*C. sporogenes*-NT) containing the empty vector were named CB0 and CS0, respectively, as negative controls. Eleven derivatives were obtained, including CB1, CB2, CB3, CB4, and CB5 for *C. butyricum*, CS1, CS2, CS3, CS4, and CS5 for *C. sporogenes*-NT, and CS3WT for wild-type *C. sporogenes* (Fig. 1A).

Western blotting analysis conducted at OD_600 _= 1.0 revealed that, among the eleven derivations, the recombinant NY-ESO-1 was efficiently expressed and secreted, appearing as bands with sizes of approximately 26 kDa and 43 kDa in the culture supernatant (S) while being minimally detected in the pellet lysate (L). The empty controls CB0 and CS0 showed no detectable NY-ESO-1 expression (Fig. 1B and C). Furthermore, derivations CB2, CB4, CS3, and CS5 exhibited strong detection signals in the culture supernatant under the influence of the P*thl*_fU, miniP*c.b*_fU, P*fdx*, and miniP*c.sp*_fU promoters, respectively.

These findings demonstrate the successful engineering of *C. sporogenes* and *C. butyricum* for the efficient overexpression and secretion of recombinant full-length NY-ESO-1, highlighting their potential as hosts for the production of this cancer vaccine candidate.

### *C. sporogenes* and *C. butyricum* secrete the adjuvant-fused NY-ESO-1, utilizing an AUU start codon

**Fig. 2:**
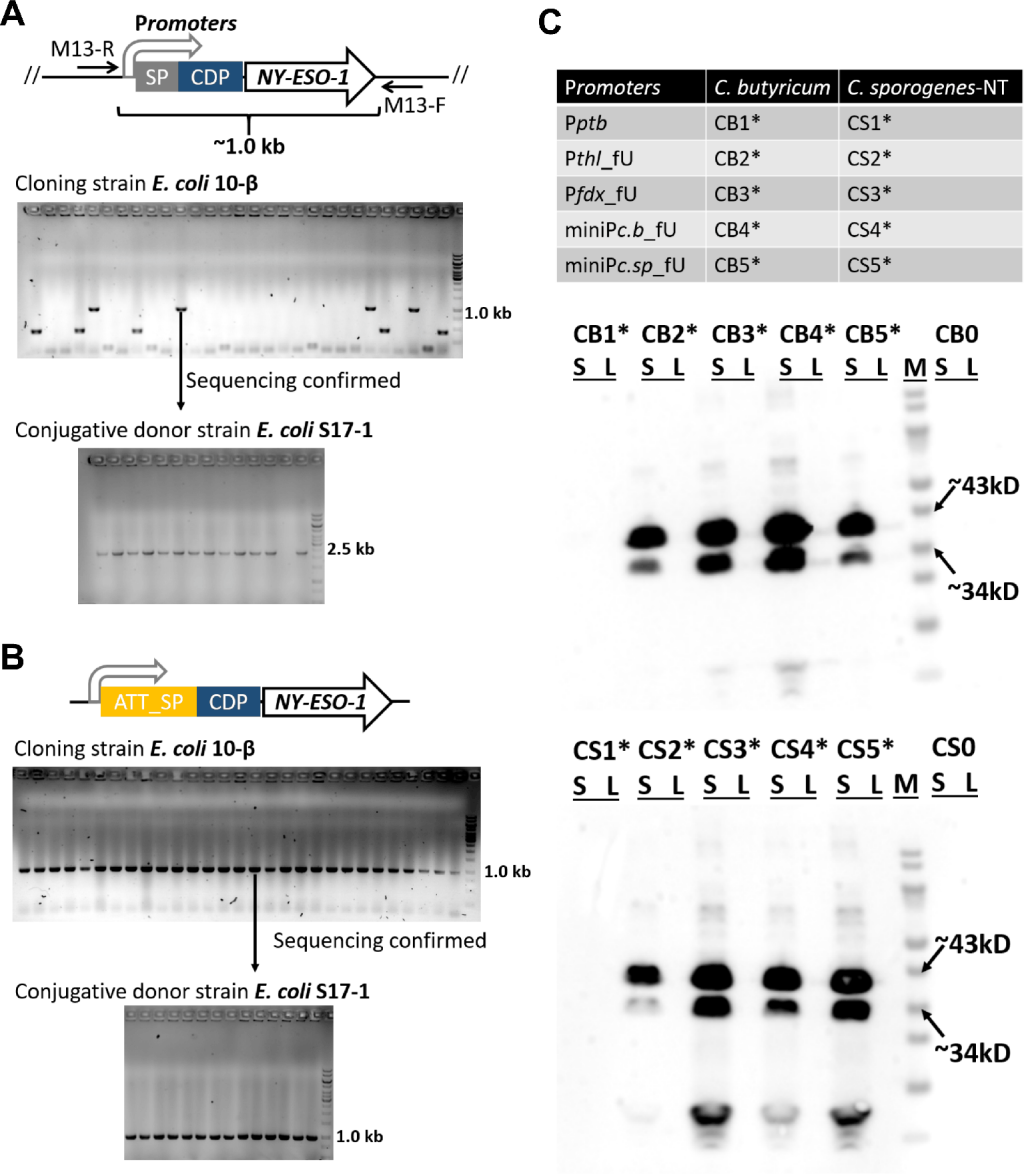
*C. sporogenes* and *C. butyricum* secrete CDP-NY-ESO-1, utilizing an AUU start codon. CDP is a reported chimeric designer peptide of *E. coli* origin. Using the primer pair M13-R/F, colony PCR was used to check the CDP-NY-ESO-1 expression plasmid with the AUG start codon (**A**) and the AUU start codon (**B**) in *E. coli* 10-β and S17-1. The P*fdx* promoter was used, and the correct PCR size was 967 bp. The GeneRuler 1-kb DNA ladder was used as the molecular weight standard. (**C**) The used CDP-NY-ESO-1 expression clostridia and Western blotting analysis of CDP-NY-ESO-1 expression in *C. butyricum* and *C. sporogenes*. S, culture supernatant; L, pellet lysate; M, PageRuler Prestained Protein Ladder

In an effort to enhance immune recognition of the NY-ESO-1 antigen, the bacterial CDP adjuvant was fused to the full-length NY-ESO-1. Initially, the five promoters (P*ptb*, P*thl*_fU, P*fdx*_fU, miniP*c.b*_fU, and miniP*c.sp*_fU) and the signal peptide, Sp*nprM3*, were ligated with the codon-optimized CDP sequence and the *NY-ESO-1* gene into the pGG2121 vector. However, in the cloning strain *E. coli* 10-β, colony PCR revealed low ligation efficiency with four correct 1.0-kb bands from 29 colonies (Fig. 2A), and Sanger sequencing confirmed random single-nucleotide polymorphism events (SNPs) in the CDP-*NY-ESO-1* region (Figure S1). Despite the identification of a sequencing-confirmed construct with the P*fdx*_fU promoter, plasmid transformation into the conjugative donor strain *E. coli* S17-1 resulted in frequent insertion sequence events (ISs) of approximately 1.5 kb in size (Fig. 2A). During the construction of a single NY-ESO-1, without the CDP fusion, no SNPs or ISs were observed. We, therefore, hypothesized that the consistent expression of CDP-fused NY-ESO-1 in *E. coli* might induce toxicity.

To address this, we reasoned that the non-AUG start codons would downregulate the expression of CDP-fused NY-ESO-1 in *E. coli*. The start codon in the Sp*nprM3* sequence was changed to AUU, designated ATT_SP, and the same construction processes were applied. Figure 2B shows that colony PCR indicated high ligation efficiency in 10-β (all correct 1.0-kb bands from 29 colonies) and rare ISs in S17-1, suggesting that the expressing toxicity of the CDP-fused NY-ESO-1 could be repressed in *E. coli* by changing the start codon.

Subsequently, ten derivations, named CB1*, CB2*, CB3*, CB4*, and CB5* for *C. butyricum*, and CS1*, CS2*, CS3*, CS4*, and CS5* for *C. sporogenes*-NT, were generated and grown until OD_600_ = 1.0. Western blotting analysis revealed that the recombinant protein of the CDP-fused NY-ESO-1, appearing as bands with sizes of approximately 34 kDa and less than 43 kDa, was expressed and secreted into the supernatant of *Clostridium* culture under the influence of the P*thl*_fU, P*fdx*_fU, miniP*c.b*_fU, and miniP*c.sp*_fU promoters. No signal was observed in the empty controls (CB0 and CS0). Notably, the use of the P*ptb* promoter in the strains CB1* and CS1* resulted in no detectable signal (Fig. 2C). These results underscore that *C. sporogenes* and *C. butyricum* can be engineered to use the AUU start codon, initiating translation and enabling the secretion of recombinant proteins, such as the bacterial adjuvant fused NY-ESO-1 antigen.

### Genome-engineered clostridia secrete recombinant NY-ESO-1 and CDP-NY-ESO-1 antigens from the PyrE loci

**Fig. 3:**
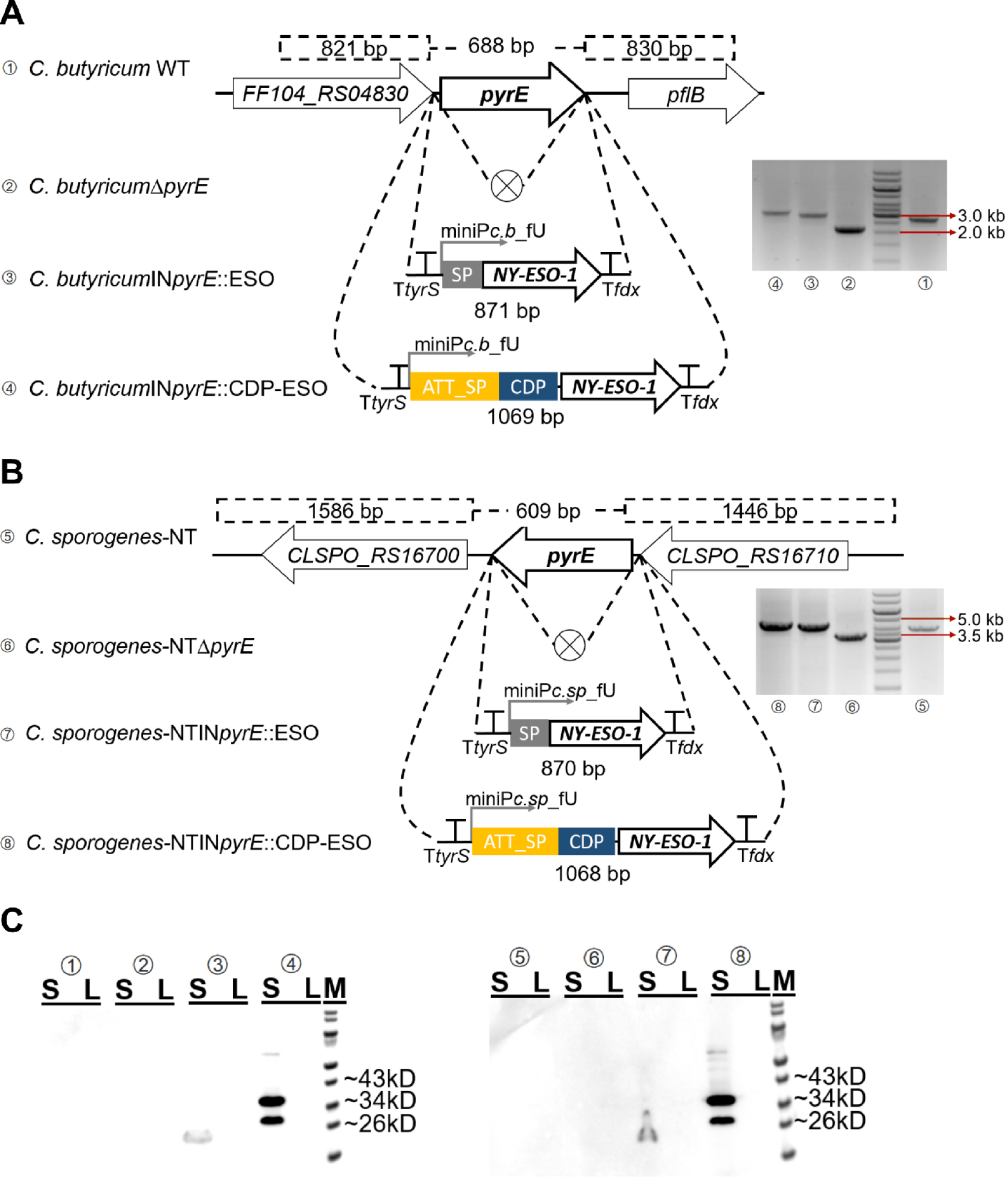
Genome-engineered clostridia secrete recombinant NY-ESO-1 and CDP-NY-ESO-1 antigens from the PyrE loci. The scheme of genome engineering in *C. butyricum* (**A**) and *C. sporogenes*-NT (**B**). Colony PCR to confirm the *pyrE* gene deletions and the integration of the NY-ESO-1 and CDP-NY-ESO-1 expression cassettes into the PyrE loci. The GeneRuler 1-kb DNA ladder was used as the molecular weight standard. (**C**) Western blotting analysis of the expression of recombinant NY-ESO-1 and CDP-NY-ESO-1 in genome-engineered clostridia. S, culture supernatant; L, pellet lysate; M, PageRuler Prestained Protein Ladder

In order to create plasmid-free clostridia for regulatory approval of COSV, the PyrE loci in the genomes of *C. butyricum* and *C. sporogenes* were replaced with high-expression cassettes carrying NY-ESO-1 and CDP-NY-ESO-1, utilizing the previously developed CRISPR-Cas12a system [31]. The introduction of uracil auxotrophy in the *pyrE*-deficient clostridia is known to ensure CBT bio-containment [16]. Consequently, the expression cassettes of miniP*c.b*_fU-*NY-ESO-1* and miniP*c.b*_fU-CDP-*NY-ESO-1* were integrated into the chromosome of *C. butyricum*, resulting in strains *C. butyricum*IN*pyrE*::ESO and *C. butyricum*IN*pyrE*::CDP-ESO (Fig. 3A). Similarly, strains *C. sporogenes*-NTΔ*pyrE*, *C. sporogenes*-NTIN*pyrE*::ESO, and *C. sporogenes*-NTIN*pyrE*::CDP-ESO were generated in this study (Fig. 3B). PCR and Sanger sequencing validated these integrations.

Considering the reported decrease in gene expression levels after chromosomal integration [19], the sample loading volume in Western blotting analysis was doubled. The results revealed the presence of recombinant NY-ESO-1 and CDP-NY-ESO-1 antigens exclusively in the culture supernatant (S) of *C. butyricum*IN*pyrE*::ESO, *C. butyricum*IN*pyrE*::CDP-ESO, *C. sporogenes*-NTIN*pyrE*::ESO, and *C. sporogenes*-NTIN*pyrE*::CDP-ESO strains (Fig. 3C). This data substantiates the successful genome engineering of *C. sporogenes* and *C. butyricum*, targeting the PyrE loci, to secrete recombinant NY-ESO-1 and CDP-NY-ESO-1 antigens without the need for antibiotic-marker plasmids.

### Genetically manipulated clostridia secrete recombinant full-length Spike_S1 and bacterial adjuvant-fused Spike_S1 antigens

**Fig. 4:**
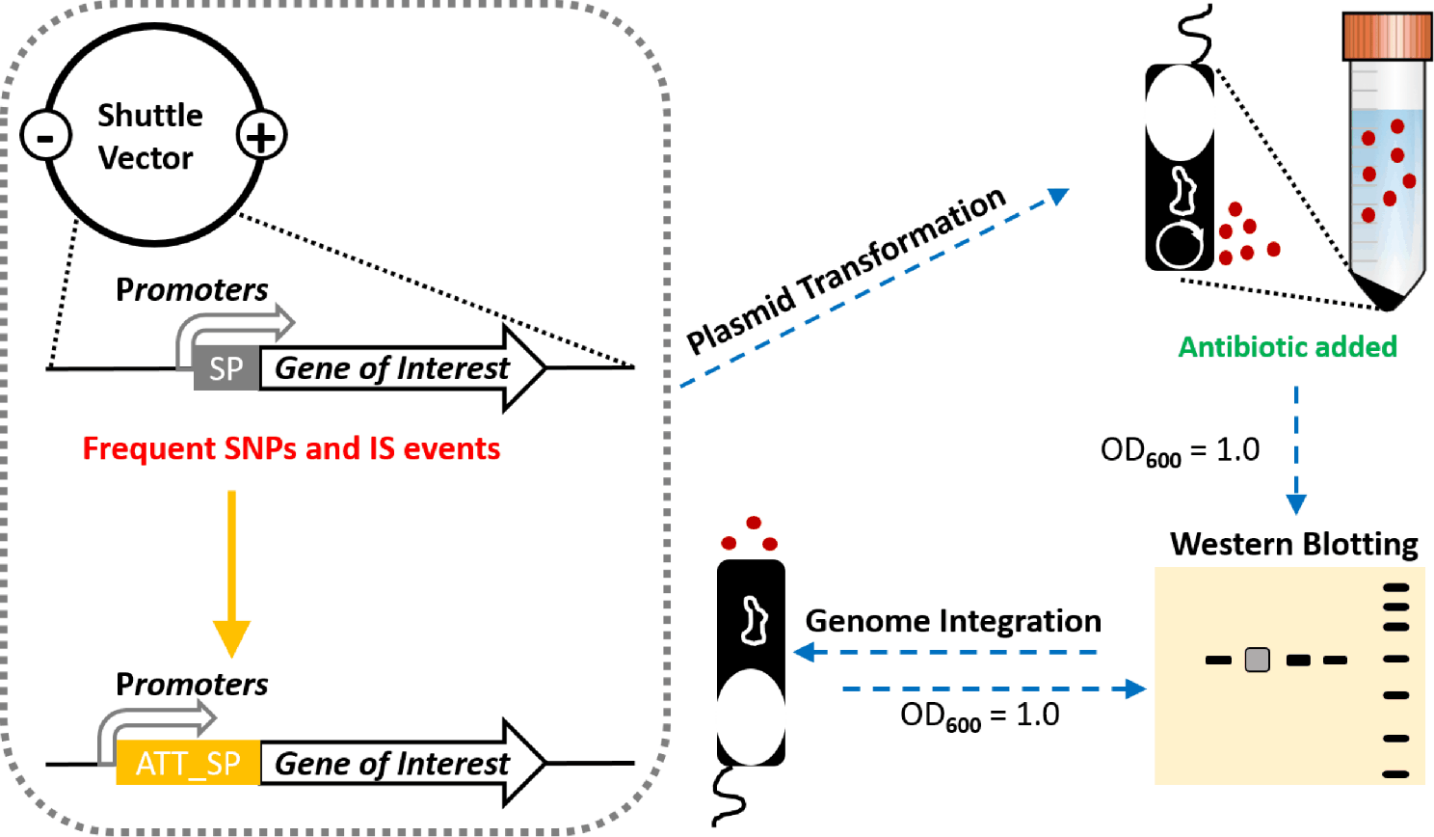
Procedure for genetic manipulations in clostridia for heterologous protein secretion. Briefly, the genes of interest, promoters, and signal peptides were ligated into *E. coli*-*Clostridium* shuttle vectors. The AUU start codon in the signal peptide would be used when difficulties (SNPs or ISs) were observed in *E. coli*. Following the transformation into clostridia, Western blotting analysis was conducted to identify the ideal expression cassette at the same growth phase. Finally, the expression cassette was integrated into the *Clostridium* chromosome by the CRISPR-Cas12a system and further validated through Western blotting analysis

To further extend the use of multiplex genetic manipulations in clostridia for heterologous protein secretion, as illustrated in Fig. 4, genes of interest were ligated with promoters and signal peptide sequences into *E. coli*-*Clostridium* shuttle vectors. During the cloning or conjugation process, when SNPs and ISs occurred, the start codon in the signal peptide sequence was modified to ATT from the conventional ATG for repression of potential toxicity to *E. coli*. Subsequently, the plasmid derivatives were transformed into clostridia, and with the presence of necessary antibiotics, Western blotting was performed to identify the most suitable expression cassette at the same growth phase (OD_600_ = 1.0). Finally, the chosen expression cassette was integrated into the PyrE loci and further validated through Western blotting analysis.

**Fig. 5:**
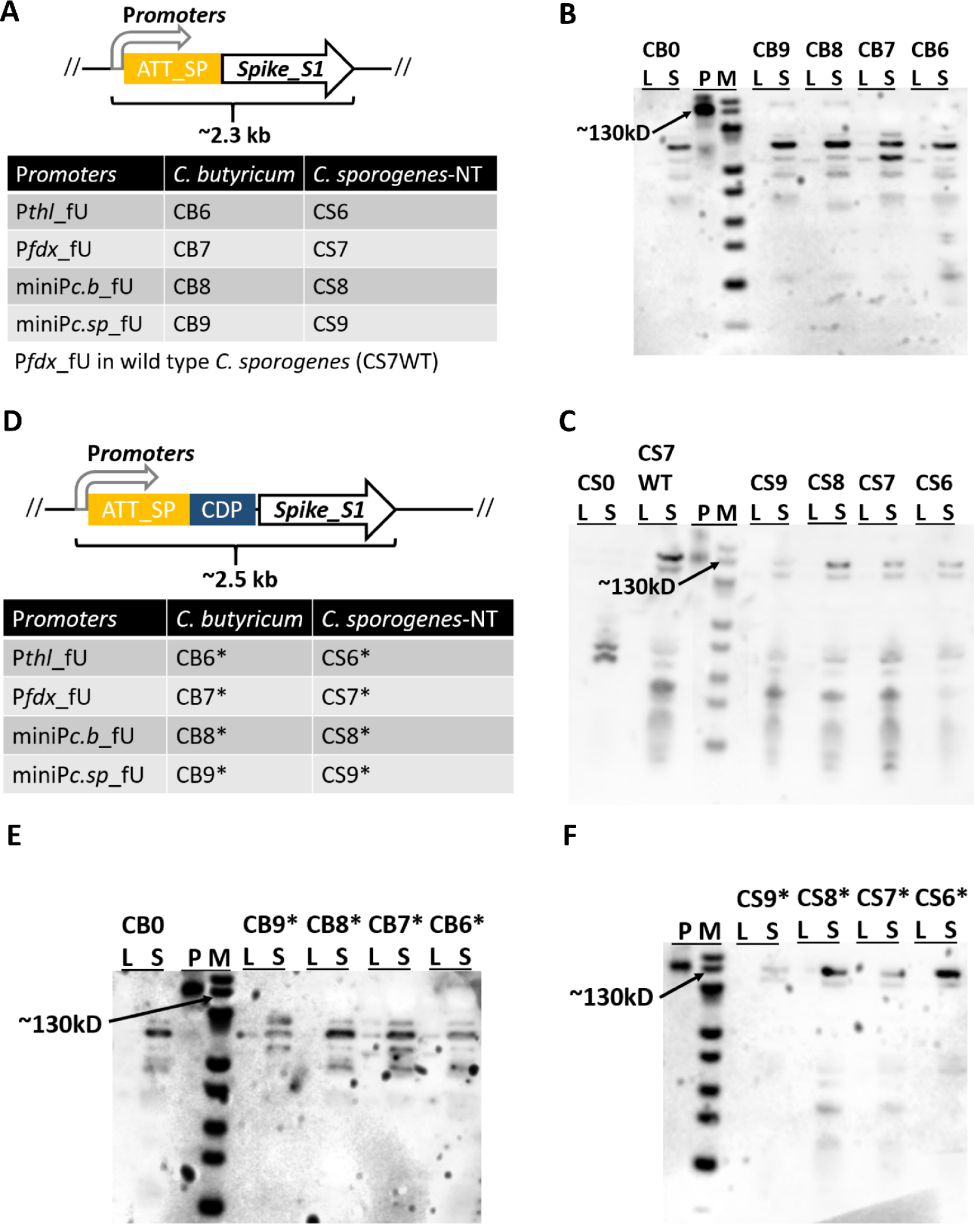
Genetically manipulated clostridia secrete recombinant SARS-CoV-2-related antigens. The schema and strain lists for the secretion of full-length Spike_S1 (**A**) and bacterial adjuvant (CDP)-fused Spike_S1 (**D**) antigens in clostridia. Western blotting analysis of the expression of recombinant full-length Spike_S1 in *C. butyricum* (**B**) and *C. sporogenes* (**C**) and the expression of recombinant CDP-Spike_S1 in *C. butyricum* (**E**) and *C. sporogenes*-NT (**F**). P, purified SARS-CoV-2 S1 protein produced in HEK293 cells; S, culture supernatant; L, pellet lysate; M, PageRuler Prestained Protein Ladder

As a proof of concept, we endeavoured to engineer *C. butyricum* and *C. sporogenes* for the secretion of SARS-CoV-2 Spike_S1 antigens. Initially, the full-length Spike_S1 sequence was codon-optimized, and challenges were encountered during the cloning process, such as limited colony growth with SNPs or the loss of the signal peptide sequence (data not shown). Therefore, ATT_SP was employed to fuse the full-length Spike_S1 sequence with four promoters (P*thl*_fU, P*fdx*_fU, miniP*c.b*_fU, and miniP*c.sp*_fU). The resultant expression plasmids were then transformed into *C. butyricum* and *C. sporogenes*, yielding nine derivatives for *C. butyricum* (CB6, CB7, CB8, and CB9) and *C. sporogenes* (CS6, CS7, CS8, and CS9 of C. sporogenes-NT, and CS7WT of wild-type *C. sporogenes*) (Fig. 5A).

Using the recombinant S1 protein produced in HEK293 cells as a positive control, Western blotting analysis confirmed the secretion of the recombinant Spike_S1 with a size of about 130 kDa [33] in strains CB6, CB8, CB9, CS6, CS7, CS8, CS9, and CS7WT. No signal of the same size was observed in the negative controls CB0 and CS0 (Fig. 5B and C). Furthermore, the bacterial adjuvant CDP was fused into the Spike_S1 sequence, resulting in eight derivatives for *C. butyricum* (CB6*, CB7*, CB8*, and CB9*) and *C. sporogenes*-NT (CS6*, CS7*, CS8*, and CS9*). In *C. butyricum*, only the P*thl*_fU promoter led to a detectable signal of the recombinant CDP-S1 protein (Fig. 5E). At OD_600_ = 1.0, all *C. sporogenes* strains secreted the recombinant CDP-S1 protein, with the P*thl*_fU and miniP*c.b*_fU promoters resulting in the strongest detection signal in *C. sporogenes* (Fig. 5F). Subsequently, the expression cassettes of miniP*c.b*_fU-S1 and miniP*c.b*_fU-CDP-S1 were integrated into the PyrE loci of the *C. sporogenes*-NT chromosome and confirmed by PCR (Figure S2) and Sanger sequencing, resulting in the *C. sporogenes*-NTIN*pyrE*::S1 and *C. sporogenes*-NTIN*pyrE*::CDP-S1 strains. Unfortunately, due to a decrease in expression, we failed to detect the signal in the culture of these two strains via Western blotting analysis, despite an increased loading volume. These data suggest that clostridia can effectively secrete recombinant SARS-CoV-2-related Spike_S1 protein through multiplex genetic manipulations.

### Secreted recombinant proteins in clostridia lack glycosylation

The lack of post-translational modifications like glycosylation may influence the utility of prokaryotic expression hosts [34]. To confirm the glycosylation status of secreted recombinant protein in clostridia, the Protein Deglycosylation Mix II (P6044, NEB) was used for the deglycosylation reaction. Followed by Western blotting analysis, samples of recombinant NY-ESO-1 protein from *C. butyricum* could not show the changes in detecting size, and a similar result was obtained in the recombinant Spike_S1 protein from *C. sporogenes* (Figure S3A). In addition, Western blotting analysis with the exchange of another Anti-SARS-CoV-2 Spike Glycoprotein S1 antibody (EPR24852-116, abcam) could only detect the recombinant SARS-CoV-2 S1 protein produced in HEK293 cells, with no detection of the recombinant Spike_S1 protein from *C. sporogenes* (Figure S3B). These results reveal a lack of glycosylation in the secreted recombinant proteins by clostridia.

## Discussion

*Clostridium* spp. stands out as a prominent component of the gut microbiota, actively interacting with host organisms and significantly contributing to human health [16]. The exploration of commensal clostridia for oral vaccination holds great promise in medical applications, presenting a novel avenue beyond the extensively studied *E. coli*, *Salmonella*, and lactic acid bacteria in prokaryotic oral vaccination research [35–37]. The primary advantage of oral vaccination lies in its potential to induce mucosal immunity, the body’s first line of defence against many pathogens entering through mucosal surfaces [38, 39]. Additionally, practical benefits include easier administration, higher patient compliance, reduced logistical costs, elimination of needle-stick injuries, possibility for self-administration, and a painless, non-invasive experience, making it particularly advantageous in resource-limited areas and for individuals with needle phobia [40]. The COSV potential is evident from the study using commensal nontoxigenic *Clostridioides difficile*. Through the overexpression of a toxin-original chimeric vaccine, this commensal strain effectively prevented infection from toxigenic *C. difficile in vivo*, highlighting the potential of leveraging commensal clostridia for innovative vaccination strategies [41].

Our work successfully executes multiplex genetic manipulations for the secretion of recombinant antigen proteins in two commensal *Clostridium* species, specifically *C. butyricum* and *C. sporogenes.* Further investigations, including the immunogenicity of these secreted antigens [42], their successful presence in intestinal conditions, and the induction of specific antibodies [43], would advance the COSV concept in two potential applications: a vaccine against cancer and an antiviral vaccine.

Previously, a promoter library was developed to regulate heterologous gene expression in *C. butyricum* and *C. sporogenes* [30]. However, using the same promoter to drive different heterologous genes could result in varying levels of protein production within cells [44]. As demonstrated here, the validated strong promoters, miniP*c.b*_fU for *C. butyricum* and miniP*c.sp*_fU for *C. sporogenes*, facilitated the most efficient production of the secreted NY-ESO-1 and CDP-fused NY-ESO-1 antigens in *C. butyricum* and *C. sporogenes*, respectively, but not for Spike_S1 antigens. Additionally, the efficiency of secreted protein production may differ depending on the combination of promoter and signal peptide sequences [45], despite the good compatibility of the *nprM3* signal peptide here in *C. butyricum* and *C. sporogenes*. To determine the most suitable expression cassette in this work, we used the same loading volume from the supernatant precipitate at the same growth phase for Western blotting analysis.

While *E. coli* has been a staple host cell for molecular biology cloning in *Clostridium* research, the persistent overexpression of heterologous proteins in *E. coli* during cloning poses challenges. During the cloning of the CDP-fused NY-ESO-1, we observed frequent SNPs within the expression cassette, potentially influencing cell survival. Additionally, the ISs occurrence in S17-1, as revealed by Sanger sequencing, includes a sequence from the IS4 family transposase within the expression cassette (Table S3). In *E. coli*, these transposable elements have been shown to play a defensive role in muting harmful genes [46].

Apart from the solitary NY-ESO-1 expression, the heterologous genes in our study may be deleterious to *E. coli*, and we encountered challenges in explaining and defining these harmful genes in *E. coli*. In this context, the signal peptide Sp*nprM3*, derived from *C. sporogenes*, was fused with all recombinant proteins to facilitate secretion in clostridia. Signal peptides from Gram-positive bacteria have been shown to function in Gram-negative bacteria [47]. Using SignalP 6.0 [48] to predict signal peptides amongst all fused genes, both gram-positive and gram-negative settings indicated outputs associated with the Sec pathway. The recombinant proteins could be deposited into the *E. coli* periplasm, and differential protein solubility may lead to variable cell fragility. Additionally, variations in recombinant protein levels may be influenced by gene contexts with different mRNA structures, stability, and codon usage [49]. Therefore, for heterologous protein secretion in clostridia, the initial choice of the classical AUG start codon is recommended to assess protein toxicity during cloning in *E. coli*.

It is well documented in eukaryotes and prokaryotes that non-AUG start codons can frequently be used for non-canonical translation initiation [50]. Previously, we reported the species-specific differences in translation initiation between clostridia and *E. coli*, and using the UUG start codon, a heterologous *sacB* gene was successfully cloned and expressed as a negative marker in *C. butyricum* [30]. Although methionine has been confirmed to commence translation initiation in eukaryotes regardless of the codon for the amino acid at the start position [51], the amino acid initiated by the non-AUG start codon is still unknown in clostridia, which might be a concern when using the non-AUG start codon for the secretion of heterologous proteins by the Sec pathway [52]. Our work demonstrates the successful use of the non-AUG start codon, AUU, for efficient translation initiation and protein secretion in clostridia. This approach offers a valuable alternative for molecular biology in clostridia, bypassing the cloning burden associated with consistent overexpression in *E. coli*.

It is known that heterologous genes in an antibiotic-based plasmid have a higher expression level than those in the chromosome of a host, due to differences in copy number [53]. In our work, cells containing the plasmid were collected with a requirement of antibiotics. Considering the challenges associated with antibiotic-based plasmid stability and the global spread of antibiotic resistance [54], strategies for plasmid stability under antibiotic-free conditions should be developed in clostridia, which have been studied in model microbes like *E. coli* and *Bacillus subtilis* [55, 56]. On the other hand, in a recent study, the potential for plasmid-DNA transfer between different *Clostridium* species (*Clostridium acetobutylicum* and *Clostridium ljungdahlii*) [57] underscores the need for comprehensive studies on chromosome integration with heterologous genes to ensure biosafety in the CBT context. To further improve heterologous gene expression from the chromosome, multi-integrations [58, 59] and rational selection of integration sites [60] can be employed in clostridia.

Glycosylation plays a crucial role in protein folding and stability, and affects the biological functions of proteins [61]. For instance, the glycosylated NY-ESO-1 protein from the yeast, *Saccharomyces cerevisiae*, has been proven to induce enhanced immune responses [62], and SARS-CoV-2 produced highly glycosylated proteins, affecting its infectivity and immune escape [63]. We confirmed a lack of glycosylation in the secreted recombinant NY-ESO-1 and Spike_S1 proteins, which may influence COSV efficacy, and thus protein targets like bacterial antigens, which do not need glycosylation, might be more appropriate for COSV. However, the immunological effects and clinical activity observed in previous phase I clinical trials of bacterial vaccines with NY-ESO-1 expression [64], as well as the protection conferred in vivo by recombinant SARS-CoV-2 proteins produced by *E. coli* BL21 [29], provide optimism for further investigations of immunological impact in vivo by the COSV strains constructed in this work. Of note, recent work presented the possibility of *E. coli*-based glycosylation machinery for producing glycoproteins [65, 66], and the continuous findings and efforts in the future might facilitate prokaryotic expression systems and bacteria-based therapy like CBT.

## Conclusions

In this study, we have demonstrated the successful secretion of recombinant antigen proteins (NY-ESO-1 and SARS-CoV-2-related Spike_S1 antigens) in *C. butyricum* and *C. sporogenes* through the implementation of multiplex genetic manipulations, encompassing both plasmid-based overexpression and chromosome-based expression strategies. The utilization of AUU start codons has proven to be a valuable solution, effectively addressing the challenges associated with cloning potentially toxic proteins in *E. coli* while enabling efficient protein secretion in clostridia. Further studies in vivo and in vitro should be necessary to confirm the efficacy of these recombinant antigens.

### Electronic supplementary material

Below is the link to the electronic supplementary material.

Supplementary Material 1

## Data Availability

All data generated or analysed during this study are included in this published article and its supplementary information files.

## Abbreviations

CDEPT: clostridia-directed enzyme prodrug therapy
CMIT: *Clostridium*-mediated cancer immunotherapy
COSV: *Clostridium* oral-spore vaccination
CBT: *Clostridium*-based bio-therapeutics
NY-ESO-1: New York esophageal squamous cell carcinoma 1
CDP: chimeric designer peptide
SNPs: single-nucleotide polymorphism events
ISs: insertion sequence events
