# Supplementary material for: Multiplex genetic manipulations in Clostridium butyricum and Clostridium sporogenes to secrete recombinant antigen proteins for oral-spore vaccination
Source: Microb Cell Fact. 2024 Apr 24;23:119. doi: 10.1186/s12934-024-02389-y (PMC11040787; doi:10.1186/s12934-024-02389-y)
Supplement: Supplementary file 1 — Supplementary Material 1 [file 12934_2024_2389_MOESM1_ESM.docx]

**Supplementary Information**

**Multiplex Genetic Manipulations in *Clostridium butyricum* and *Clostridium sporogenes* to Secrete Recombinant Antigen Proteins for Oral-Spore Vaccination**

Yanchao Zhang ^1, *^, Tom S. Bailey ^1, 2^, Philip Hittmeyer ^1, 3^, Ludwig Dubois ^1^, Jan Theys ^1^, Philippe Lambin ^1, *^

^1^ The M-Lab, Department of Precision Medicine, GROW - Research Institute for Oncology and Reproduction, Maastricht University, 6229 ER Maastricht, the Netherlands

^2^ Department of Cell Biology–Inspired Tissue Engineering, MERLN Institute for Technology-Inspired Regenerative Medicine, Maastricht University, 6229 ER Maastricht, the Netherlands

^3^ LivingMed Biotech BV, Clos Chanmurly 13, 4000 Liège, Belgium

* To whom correspondence should be addressed. Email: [yanchao.zhang@maastrichtuniversity.nl](mailto:yanchao.zhang@maastrichtuniversity.nl) & [philippe.lambin@maastrichtuniversity.nl](mailto:philippe.lambin@maastrichtuniversity.nl)

**Table S1.** Primers used in the study.

**Table S2.** Sequences of gBlock fragments codon-optimized and synthesized by Integrated DNA Technologies.

**Table S3.** The IS4 sequence in the CDP-*NY-ESO-1*.

**Figure S1.** Sanger sequencing confirmed SNPs in the CDP-*NY-ESO-1* region.

**Figure S2.** The expression cassettes of recombinant SARS-CoV-2-related antigens were integrated into the PyrE loci of *C. sporogenes*-NT.

**Figure S3.** Secreted recombinant proteins in clostridia lack glycosylation.

**Table S1. Primers used in the study.** Restriction sites are underlined.

| **Primers** | **5’ - 3’ Sequences** | **Description** |
| --- | --- | --- |
| M13-R | CAGGAAACAGCTATGACCG | Universal primer pair of colony PCR for derivative vectors of pGG2121 |
| M13-F | GTAAAACGACGGCCAGTG |  |
| CDP_ESO-F-BsaI | GGTCTCATAGTCAAGCTGAAGGTAGAG | Amplifying the NY-ESO-1 fragment for CDP fusion (CDP_ESO) |
| ESO-R-BsaI | GGTCTCTGTCTTTATCTTCTTTGACCTGAT |  |
| ATT_SP-F-BsaI | GGTCTCGACATATTAAAAGTAAAAAATTATTAGCTACAGTG | Amplifying the ATT_SP-CDP fragment |
| CDP-R-BsaI | GGTCTCAACTACCTGAACCACTAC |  |
| S1-R-BsaI | GGTCTCAGTCTTTAACGTGCTCTTCTAGG | Amplifying the ATT_SP-S1 fragment with the primer ATT_SP-F-BsaI |
| CDP_S1-F-BsaI | GGTCTCATAGTCAGTGTGTTAATCTTACAAC | Amplifying the S1 fragment for CDP fusion with the primer S1-R-BsaI (CDP_S1) |
| uni_IN-F-BsaI | GGTCTCGTGGAGGATAATCAATCGTCCCTTC | Amplifying the fragments of the gene expression cassette from the derivative vectors of pGG2121 |
| uni_IN-R-BsaI | GGTCTCATCAGAACGGCGCGCC |  |
| Tc.bPyrE-F-BsmBI | CGTCTCGAGATCATCAACATCTCCTTGGCTCT | Amplifying the target fragment for *C. butyricum* PyrE loci |
| Tc.bPyrE-R-BsmBI | CGTCTCGAATTAAAGAGCCAAGGAGATGTTG |  |
| c.bPyrE-U-F-AatII | GAGAGGACGTCGATGTAGGAATTCTTGGAGG | Amplifying the upstream fragment for *C. butyricum* PyrE loci |
| c.bPyrE-U-R-BsaI | GGTCTCCTGTAGTTAACTTCAAACTTTTTTATTAAATAATTCC |  |
| c.bPyrE-D-F-BsaI | GGTCTCGTACAATATATAGGGAAAAATAAGGTGAT | Amplifying the downstream fragment for *C. butyricum* PyrE loci |
| c.bPyrE-D-R-SalI | GCGCGTCGACAGTCACTTACTTCATCCCATAAC |  |
| c.bPyrE-F | TGCTGTGGCCCTGAAGTTATGA | Colony PCR for *C. butyricum* PyrE loci |
| c.bPyrE-R | ACTTCATATCCATAAGCCTTAGCTGCA |  |
| TspPyrE-F-BsmBI | CGTCTCGAGATGCACATTGACAATATCTATTACT | Amplifying the target fragment for *C. sporogenes* PyrE loci |
| TspPyrE-R-BsmBI | CGTCTCGAATTAGTAATAGATATTGTCAATGTGCATCT |  |
| spPyrE-U-F-AatII | GAATGGACGTCAAGACATACACCAAGCT | Amplifying the upstream fragment for *C. sporogenes* PyrE loci |
| spPyrE-U-R-BsaI | GGTCTCGTCCATTTAAAAATAAGGAGTGTCT |  |
| spPyrE-D-F-BsaI | GGTCTCTCTGATTAAATAATTCCCCTTATTTCTT | Amplifying the downstream fragment for *C. sporogenes* PyrE loci |
| spPyrE-D-R-SalI | GCGAGTCGACCAAATTAGAAGGAAAAGGAA |  |
| spPyrE-F | CTCATCTCTATCATTTATATGAACATTAA | Colony PCR for *C. sporogenes* PyrE loci |
| spPyrE-R | AAAATGACCTTAGGTGGTACTT |  |

**Table S2. Sequences of gBlock fragments codon-optimized and synthesized by Integrated DNA Technologies.** The open reading frames are highlighted in bold. The *nprM3* signal peptide sequence is highlighted in red. Restriction sites are underlined.

| gBlock fragments | 5’ - 3’ Sequences |
| --- | --- |
| SP-NY-ESO-1 | GGTCTCGACAT**ATGAAAAGTAAAAAATTATTAGCTACAGTGCTAAGTGCTGTAATCACTCTTTCTACTGTTTCTGCAGTTTATGCTCAAGCTGAAGGTAGAGGAACTGGAGGTAGTACTGGAGATGCTGATGGTCCAGGTGGTCCTGGAATTCCAGATGGACCAGGTGGAAATGCTGGAGGACCTGGTGAGGCAGGTGCTACAGGAGGAAGAGGTCCTAGAGGTGCTGGAGCTGCAAGAGCATCTGGACCTGGAGGAGGAGCACCTAGAGGACCACATGGAGGTGCAGCTTCAGGTTTAAATGGATGTTGTAGATGTGGTGCTAGAGGTCCAGAAAGTAGATTACTTGAATTTTATTTAGCTATGCCATTTGCTACTCCAATGGAAGCAGAACTTGCAAGAAGAAGTTTAGCACAAGATGCACCTCCACTTCCTGTACCAGGAGTATTATTAAAGGAATTTACAGTTTCTGGTAATATTCTTACTATAAGACTTACTGCAGCTGATCATAGACAATTACAATTATCAATTAGTTCTTGTTTACAACAACTTTCTTTACTTATGTGGATAACTCAATGTTTTCTTCCAGTATTTTTAGCTCAACCACCATCAGGTCAAAGAAGATAA**AGACAGAGACC |
| SP-CDP | GGTCTCGACAT**ATGAAAAGTAAAAAATTATTAGCTACAGTGCTAAGTGCTGTAATCACTCTTTCTACTGTTTCTGCAGTTTATGCTGATAATAATAGTCTTTCTCAAGAAGTACAAAATGGTTCAAATCATTTAGAAAATAATCAATCACAATCTAATGGAGGAGGTAGTGATAGTGCATTAAGTTTATCATCAAAAACAGCTGCATTAGCTGCTGCAACAACTGTAAATGATGGATCAGATGGTGCAACATCATCAGCTGTTGGTGGTAGTGGTTCAGGTAGT**TGAGACC |
| SP-Spike_S1 | GGTCTCAACAT**ATGAAAAGTAAAAAATTATTAGCTACAGTGCTAAGTGCTGTAATCACTCTTTCTACTGTTTCTGCAGTTTATGCTCAGTGTGTTAATCTTACAACCAGAACTCAATTACCCCCTGCATACACTAATTCTTTCACACGTGGAGTATACTACCCTGACAAAGTTTTCAGATCCTCAGTTTTACATTCAACTCAGGACTTGTTCTTACCTTTCTTTTCCAATGTTACTTGGTTCCATGCTATACATGTGTCTGGGACCAATGGTACTAAGAGGTTTGATAACCCTGTACTACCATTTAATGATGGAGTTTATTTTGCTTCCACTGAGAAGTCTAACATAATAAGAGGCTGGATTTTTGGTACTACTTTAGATTCTAAGACCCAGTCCCTACTTATTGTTAATAACGCTACTAATGTAGTTATTAAAGTATGTGAATTTCAATTTTGTAATGATCCATTTTTGGGTGTATATTACCACAAAAACAACAAAAGTTGGATGGAAAGTGAGTTCAGAGTTTATTCTAGTGCTAATAATTGCACTTTTGAATATGTGTCTCAGCCTTTTCTTATGGACCTTGAAGGAAAACAGGGTAATTTTAAAAATCTTAGGGAATTTGTGTTTAAGAATATTGATGGTTATTTTAAAATTTATTCTAAGCACACTCCTATTAATTTAGTGAGAGATCTTCCTCAGGGTTTTTCAGCTTTAGAACCATTGGTAGATTTGCCAATAGGTATTAACATAACTAGGTTTCAAACTTTACTTGCTTTACATAGAAGTTATTTGACTCCTGGTGATTCTTCTTCAGGTTGGACAGCTGGTGCTGCAGCTTATTATGTGGGTTATCTTCAACCTAGGACTTTTCTATTAAAATATAATGAAAATGGAACCATTACAGATGCTGTAGACTGTGCACTTGACCCTTTATCAGAAACAAAGTGTACTTTGAAATCATTCACTGTAGAAAAAGGAATTTATCAAACTTCTAACTTTAGAGTTCAACCAACAGAATCTATTGTTAGATTTCCTAATATTACAAACTTGTGCCCTTTTGGTGAAGTTTTTAACGCCACCAGATTTGCATCTGTTTATGCTTGGAACAGGAAGAGAATTAGCAACTGTGTTGCAGATTATTCTGTGCTATATAATTCAGCATCATTTTCAACTTTTAAGTGTTATGGAGTGTCTCCTACTAAATTAAATGATCTTTGCTTTACTAATGTTTATGCAGATTCATTTGTAATTAGAGGTGATGAAGTAAGACAAATAGCTCCAGGGCAAACTGGAAAGATTGCTGATTATAATTATAAATTACCAGATGATTTTACAGGCTGCGTTATAGCTTGGAATTCTAACAATCTTGATTCTAAGGTTGGTGGTAATTATAACTACCTATATAGATTGTTTAGGAAGTCTAATCTAAAACCTTTTGAGAGAGATATTTCAACTGAAATATATCAGGCCGGTAGCACACCTTGTAATGGAGTTGAAGGTTTTAATTGTTACTTTCCTTTACAATCTTATGGTTTCCAACCCACTAATGGTGTTGGTTACCAACCATACAGAGTAGTAGTACTTTCTTTTGAACTTCTACATGCACCAGCAACTGTTTGTGGACCTAAAAAGTCTACTAATTTGGTTAAAAACAAATGTGTAAATTTCAACTTCAATGGTTTAACAGGCACAGGTGTTCTTACTGAGTCTAACAAAAAGTTTTTGCCTTTCCAACAATTTGGCAGAGACATTGCTGACACTACTGATGCTGTACGTGATCCACAGACACTTGAGATTCTTGACATTACACCATGTTCTTTCGGTGGTGTTAGTGTTATAACACCAGGAACAAATACTTCTAACCAGGTTGCTGTTCTTTATCAGGATGTTAACTGCACAGAAGTACCTGTTGCTATTCATGCAGATCAACTTACTCCTACTTGGAGAGTATATTCTACAGGTTCTAATGTATTTCAAACACGTGCAGGCTGTTTAATAGGGGCTGAACATGTTAACAACTCTTATGAGTGTGACATACCCATTGGTGCAGGTATATGCGCTAGTTATCAGACTCAGACTAATTCTCCTAGAAGAGCACGTTAA**AGACTGAGACC |

**Table S3. The IS4 sequence in the CDP-*NY-ESO-1*.** The insertion sequence is highlighted in red.

| 5’ - 3’ Sequences |
| --- |
| CCCGCATTAAATTACTTTAAAATTAATAAAAACATGGGTAAAATATAAATCGTATAAAGTTGTGTAATTTTTAAGGAGGTGTGTTACATATGAAAAGTAAAAAATTATTAGCTACAGTGCTAAGTGCTGTAATCACTCTTTCTACTGTTTCTGCAGTTTATGCTGATAATAATAGTCTTTCTCAAGAAGTACAAAATGGTTCAAATCATTTAGAAAATAATCAATCACAATCTAATGGAGGAGGTAGTGATAGTGCATTAAGTTTATCATCAAAAACAGCTGCATTAGCTGCTGCAACAACTGTAAATGATGGATCAGATGGTGCAACATCATCAGCTGTTGGTGGTAGTGGTTCAGGTAGTCAAGCTGAAGGTAGAGGAACTGGAGGTAGTACTGGAGATGCTGATGGTCCAGGTGGTCCTGGAATTCCAGATGGACCAGGTGGAAATGCTGGAGGACCTGGTGAGGCAGGTGCTACAGGAGGAAGAGGTCCTAGAGGTGCTGGAGCTGCAAGAGCATCTGGACCTGGAGGAGGAGCACCTAGAGGACCACATGGAGGTGCAGCTTCAGGTTTAAATGGATGTTGTAGATGTGGTGCTAGAGGTCCAGAAAGTAGATTACTTGAATTTTATTTAGCTATGCCATTTGCTACTCCAATGGAAGCAGAACTTGCAAGAAGAAGTTTAGCACAAGATGCACCTCCACTTCCTGTACCAGGAGTATTATTAAAGGAATTTACAGTTTCTGGTAATATTCTTACTATAAGACTTACTGCAGCTGATCATAGACAATTACAATTATCAATTAGTTCTTGTTTACAACAACTTTCTTTACTTATGTGGATAACTCAATGTTTTCTTCCAGTATTTTTAGCTCAACCACCATCAGGTCAAAGAAGATAAAGACATGCAAGCTTGGCACTGATGAATCCCCTAATGATTTTTATCAAAATCATTAAGTTAAGGTAGATACACATCTTGTCATATGATCAAATGGTTTCGCCAAAAATCAATAATCAGACAACAAAATGTGCGAACTCGATATTTTACACGACTCTCTTTACCAATTCTGCCCCGAATTACACTTAAAACGACTCAACAGCTTAACGTTGGCTTGCCACGCCTTACTTGACTGTAAAACTCTCACTCTTACCGAACTTGGCCGTAACCTGCCAACCAAAGCGAGAACAAAACATAACATCAAACGAATCGACCGATTGTTAGGTAATCGTCACCTCCACAAAGAGCGACTCGCTGTATACCGTTGGCATGCTAGCTTTATCTGTTCGGGCAATACGATGCCCATTGTACTTGTTGACTGGTCTGATATCCGTGAGCAAAAACGGCTTATGGTATTGCGAGCTTCAGTCGCACTACACGGTCGTTCTGTTACTCTTTATGAGAAAGCGTTCCCGCTTTCAGAGCAATGTTCAAAGAAAGCTCATGACCAATTTCTAGCCGACCTTGCGAGCATTCTACCGAGTAACACCACACCGCTCATTGTCAGTGATGCTGGCTTTAAAGTGCCATGGTATAAATCCGTTGAGAAGCTGGGTTGGTACTGGTTAAGTCGAGTAAGAGGAAAAGTACAATATGCAGACCTAGGAGCGGAAAACTGGAAACCTATCAGCAACTTACATGATATGTCATCTAGTCACTCAAAGACTTTAGGCTATAAGAGGCTGACTAAAAGCAATCCAATCTCATGCCAAATTCTATTGTATAAATCTCGCTCTAAAGGCCGAAAAAATCAGCGCTCGACACGGACTCATTGTCACCACCCGTCACCTAAAATCTACTCAGCGTCGGCAAAGGAGCCATGGATTCTAGCAACTAACTTACCTGTTGAAATTCGAACACCCAAACAACTTGTTAATATCTATTCGAAGCGAATGCAGATTGAAGAAACCTTCCGAGACTTGAAAAGTCCTGCCTACGGACTAGGCCTACGCCATAGCCGAACGAGCAGCTCAGAGCGTTTTGATATCATGCTGCTAATCGCCCTGATGCTTCAACTAACATGTTGGCTTGCGGGCGTTCATGCTCAGAAACAAGGTTGGGACAAGCACTTCCAGGCTAACACAGTCAGAAATCGAAACGTACTCTCAACAGTTCGCTTAGGCATGGAAGTTTTGCGGCATTCTGGCTACACAATAACAAGGGAAGACTTACTCGTGGCTGCAACCCTACTAGCTCAAAATTTATTCACACATGGTTACGCTTTGGGAAATTAGAGGGTTA |

**Figure S1. Sanger sequencing confirmed SNPs in the CDP-*NY-ESO-1* region**.

**
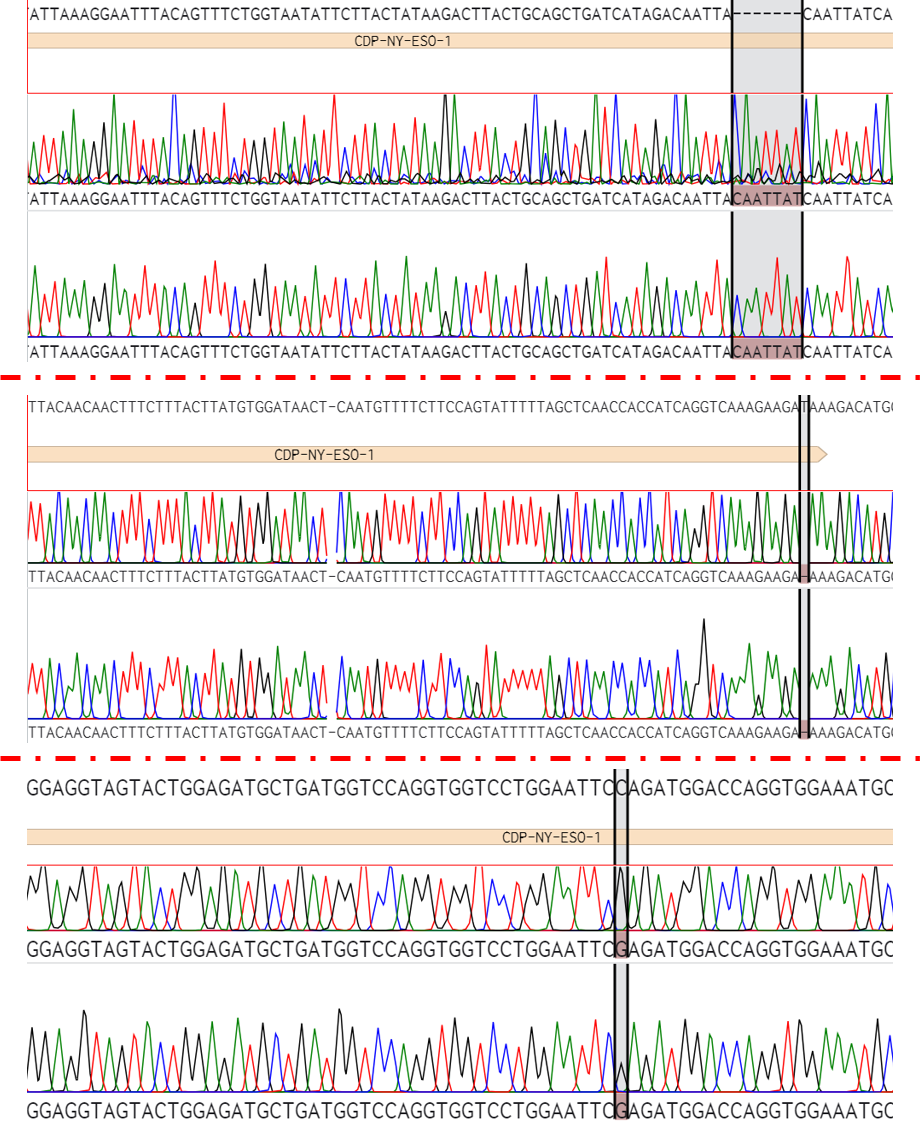
**

**Figure S2. The expression cassettes of recombinant SARS-CoV-2-related antigens were integrated into** **the PyrE loci of *C. sporogenes*-NT**. Colony PCR to confirm the *pyrE* gene deletion and the integration of the Spike_S1 and CDP-Spike_S1 expression cassettes into the PyrE loci. The GeneRuler 1-kb DNA ladder was used as the molecular weight standard., *C. sporogenes*-NT; , *C. sporogenes*-NTΔ*pyrE*; , *C. sporogenes*-NTIN*pyrE*::*S1*; , *C. sporogenes*-NTIN*pyrE*::CDP-*S1*.





**Figure S3. Secreted recombinant proteins in clostridia lack glycosylation.** (A) Deglycosylated by the Protein Deglycosylation Mix II (P6044, NEB), samples of recombinant NY-ESO-1 protein from the culture supernatant of *C. butyricum* (1, non-treated; 2, treated) and the recombinant Spike_S1 protein from the culture supernatant of *C. sporogenes* (3, non-treated; 4, treated) were conducted through Western blotting analysis. (B) Using the Anti-SARS-CoV-2 Spike Glycoprotein S1 antibody (EPR24852-116, abcam), Western blotting analysis was conducted to detect the recombinant SARS-CoV-2 S1 protein produced in HEK293 cells (P) and the recombinant Spike_S1 protein from the culture supernatant of *C. sporogenes* (3).

**

**
